# Video-assisted thoracoscopic surgery versus muscle-sparing thoracotomy for non-small cell lung cancer: a systematic review and meta-analysis

**DOI:** 10.1186/s12893-019-0618-1

**Published:** 2019-10-15

**Authors:** Zihuai Wang, Long Pang, Jiexi Tang, Jiahan Cheng, Nan Chen, Jian Zhou, Lunxu Liu

**Affiliations:** 1 Department of Thoracic Surgery, West China Hospital, Sichuan University, No. 37, Guoxue Alley, Chengdu, 610041 Sichuan China; 2 West China School of Medicine, Sichuan University, No. 37, Guoxue Alley, Chengdu, 610041 Sichuan China

**Keywords:** Non-small cell lung cancer, Video-assisted thoracoscopic surgery, Muscle-sparing thoracotomy

## Abstract

**Background:**

It has been widely accepted that video-assisted thoracoscopic surgery (VATS) lobectomy is superior to conventional open thoracotomy lobectomy in many aspects. However, the direct comparison between VATS and Muscle-sparing thoracotomy (MST) has not been widely conducted. We aimed to compare the perioperative outcomes in non-small cell lung cancer (NSCLC) patients following VATS and MST.

**Methods:**

PubMed, EMBASE, the Cochrane Library and Web of Science were searched for relevant studies. The retrieval time was up to April 24, 2019. Studies investigating the comparison of video-assisted thoracoscopy and muscle-sparing thoracotomy were included in our meta-analysis. Odds ratio and mean differences with 95% confidential interval were applied to determine the effectiveness of dichotomous or continuous variables respectively.

**Results:**

A total of 10 studies were included with 1514 patients. Compared with MST, the incidence of postoperative complications in VATS [OR = 0.54; 95%CI(0.4, 0.73); *P* < 0.001] and the hospital stay [MD = -1.5; 95%CI(− 2.28, − 0.73); *P* = 0.0001] decreased significantly, chest tube drainage time [MD = -0.71; 95%CI(− 1.18, − 0.24); *P* = 0.003] were shorter and the intraoperative blood loss [MD = − 43.87; 95%CI(− 73.66, − 14.08); *P* = 0.004] were less in VATS group. VATS also showed a relatively longer operative time [MD = 17.11; 95%CI(2.38, 31.85); *P* = 0.02]. However, no significant differences were observed in numbers of resected lymph nodes, postoperative mortality, postoperative pneumonia and postoperative bleeding.

**Conclusion:**

Compared with MST, VATS was associated with lower incidence of postoperative complications, shorter length of hospital stay, less intraoperative blood loss and less chest tube drainage, which showed that VATS was a comparable method to MST. Meanwhile, these results should be further conformed by more randomized control trials.

## Background

Lung cancer is the most commonly diagnosed cancer around the world and the leading cause of cancer death, with the highest mortality rate among men and the second mortality rate among women [1, 2]. Every year, 1.6 million new cases are diagnosed globally [3]. Additionally, prognosis of lung cancer is poor—the 5-year survival rate for lung cancer is only 18% according to NCCN guideline [4, 5].

Surgery still remains the most commonly used therapy for early stage lung cancer patients [6]. Posterolateral thoracotomy (conventional thoracotomy) and muscle sparing thoracotomy(MST) [7–9] were thought to be the main method for a long time. Comparing with conventional thoracotomy, MST had better postoperative outcomes [10]. However, with the rapid development of technology, video-assisted thoracoscopic surgery (VATS) has been widely validated in recent years [11, 12]. Minimal invasive surgery has potential superiorities of optimizing surgical indications, including safety, feasibility, less invasiveness, and a better quality of life [13]. Yet, a direct comparison between the effects of MST and VATS is still vacant.

Via a meta-analytic approach, the aim of this study is to compare these two methods in terms of perioperative outcomes.

## Methods

### Search strategy

No previous protocol has been conducted for this review. PubMed, EMBASE, the Cochrane Library and Web of Science were independently searched by two reviewers (Long Pang and Zihuai Wang) to identify potentially eligible literature up to April 23, 2019 with the following search items: (((((vats) OR video assisted thoracic surgery) OR video assisted thoracoscopic) OR minimal invasive thoracic)) AND ((((muscle sparing thoracotomy) OR muscle sparing thoracic surgery) OR muscle sparing thoracoscopic) OR muscle sparing). Only English studies were included in our analysis. A third reviewer drew the conclusion when any disagreement appeared. All potential eligible studies were manually searched to find possible relevant publications.

### Study selection

The following criteria has been set before article collection for inclusion: 1) patients with NSCLC proven pathologically; 2) compared the effects between VATS and MST; 3) RCT or cohort studies with acceptable methodological quality. The studies were excluded meeting any of the following criteria: 1) conference abstracts, reviews, letters, book chapters, animal experiments or case reports; 2) baseline characters were incomplete; 3) insufficient data for meta-analysis.

### Data collection

Data collection was independently conducted by 2 reviewers. The extracted and summarized data include: first author, publication year, study design, gender of included patients, sample size and patient staging. In addition, we analyzed the outcomes of intraoperative data, postoperative complications, length of hospital stay and chest tube drainage.

### Quality assessment

The quality of included RCT was assessed by Jadad scale [14], which contains randomization (0–2 points), blindness of the studies (0–2 points), and withdrawals (0–1 points). Studies with a score higher than three points were defined as high quality. The included non-randomized articles were assessed by Newcastle-Ottawa Scale [15]. NOS comprises three perspectives with a maximum of 9 stars when assessing non-randomized control trials. Studies with scored 0 to 3, 4 to 6 and 7 to 9 were taken as low medium and high quality, respectively.

### Statistical analysis

Review Manager V5.3 (The Cochrane Collaboration, Software Update, Oxford, UK) was used to extract, pool and analyze data. Dichotomous outcomes were evaluated by odds ratio (OR) and a corresponding 95% confidence intervals (CI). The curative effect in continuous variables was expressed as mean difference (MD) with corresponding 95% confidence intervals (CI). The results were identified as statistically significant when the *P* value less than 0.05. Statistical heterogeneity was assessed by the I^2^ statistics. If I^2^<50%, it represents no statistically significant heterogeneity exists across studies and the fixed-effects model would be used otherwise the randomized-effects model would be applied. Sensitivity analysis was performed by sequential removal of each study. Funnel plots generated by Review Manager V5.3 were used to estimate the potential publication bias in included articles. All *P* values were two-sided. A significant difference was defined as *P* < 0.05.

## Results

### Selection process

**Fig. 1:**
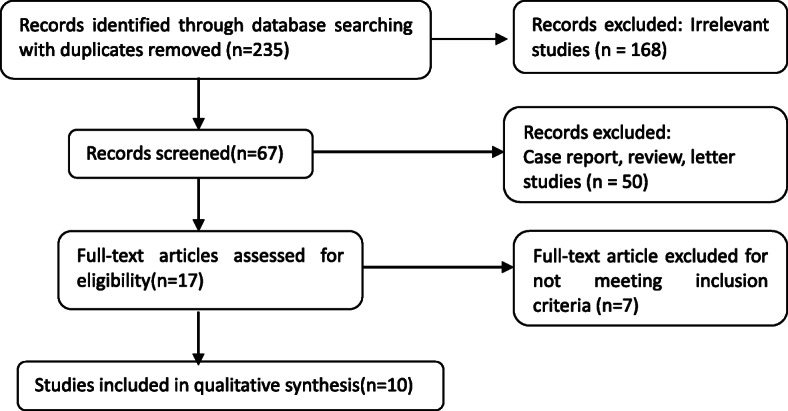
The PRISMA flow diagram of literature retrieval

In total, 235 papers were screened by the initial database search. After reviewing the abstract, 218 articles were excluded. Another 17 papers were reviewed in full text. Finally, a total of 10 eligible studies [16–25] with 1514 patients were included in this meta-analysis after fully evaluation. Although slight differences exist in VATS and MST among institutions. VATS surgery was all presented with a video camera inserted into the pleural space without rib spreading. And Muscle-sparing thoracotomy was all described as preserving related muscles. Latissimus dorsi and serratus anterior were retracted or split in the direction of the fibers. After detailed screening, the entire perioperative management of each article is ensured to be comparable. Figure 1 shows the process of article selection.

### Study characteristics and risk of bias assessment

All included articles were published before April 1st, 2019. Among the 1514 patients, 711(46.7%) of them underwent VATS, 803(53.3%) of them underwent MST. There were 1 RCT, 3 prospective studies and 5 retrospective studies. Mostly, the studies were composed of early stage patients. Three articles underwent propensity score matching [17, 19, 22]. Table 1 shows the detailed characteristics of included studies. The quality of included studies was assessed by Jadad score for the RCT and NOS for the cohort studies. In Kirby’s work, the method of randomization was not mentioned nor did the withdrawal or blindness of the patients [18]. Therefore, it was evaluated as low quality with only one score. Table 2 showed the quality assessment result of all cohort studies [16, 17, 19–24].

**Table 1:** Characteristics of the included clinical trials

Study	Year	Country	Study design	Gender (M/F)	Sample size VATS/MST	Staging	Propensity score matching	Quality assessment
Giudicelli	1994	France	Prospective	50/17	44/23	Pathologic Stage I to stage IV	Unmatched	NOS: 6
Kirby	1995	USA	RCT	26/35	30/31	Clinical stage I	Unmatched	Jadad: 1
Nomori	2001	Japan	Prospective	44/22	33/33	Clinical stage I	Unmatched	NOS: 8
Wang	2010	China	Retrospective	175/141	121/195	Pathologic Stage I to stage IV	Unmatched	NOS: 6
Ilonen	2013	Finland	Retrospective	125/107	116/116	Clinical stage I	Matched	NOS: 7
Erus	2014	Turkey	Prospective	35/20	25/30	Clinical stage I	Unmatched	NOS: 8
Kuritzky	2015	USA	Retrospective	45/75	60/60	Clinical stage I	Matched	NOS: 8
Usuda	2017	Japan	Retrospective	46/41	25/62	Clinical Stage I to stage III	Unmatched	NOS: 6
Zhao	2017	China	Retrospective	250/232	241/241	Clinical stage I	Matched	NOS: 8
Menna	2018	Italy	Retrospective	18/10	16/12	Clinical Stage I	Unmatched	NOS: 7

*Abbreviations*: *non-RCT* non-randomized controlled trial, *RCT* randomized controlled trial, *VATS* video-assisted thoracoscopic surgery, *MST* muscle-sparing thoracotomy, *NOS* Newcastle-Ottawa Scale

**Table 2:** Risk of bias assessment of included cohort studies

	Selection				Comparability	Outcome			Total score
	Exposed cohort	Non-exposed cohort	Ascertainment of exposure	Outcome of interest		Assessment of outcome	Length of follow-up	Adequacy of follow up	
Asamura	☆	☆	☆	☆	☆	☆	☆	☆	8
Chen	☆	☆	☆	☆	☆	☆	–	–	6
Guerrera	☆	☆	☆	☆	☆	☆	☆	☆	8
Hu	☆	☆	☆	☆	☆	☆	☆	–	7
Ichinose	☆	☆	☆	☆	–	☆	☆	☆	7
Ilic	☆	☆	☆	☆	☆	☆	–	–	6
Legras	☆	☆	☆	☆	–	☆	☆	☆	7
Li (case control)	☆	☆	☆	☆	☆	☆	–	–	8
Li (combined)	☆	☆	☆	☆	☆	☆	☆	☆	8
Liu	☆	☆	☆	☆	☆	☆	–	–	6
Luo	☆	☆	☆	☆	☆	☆	☆	–	7
Misthos	☆	☆	☆	☆	☆	☆	–	–	6
Misthos	☆	☆	☆	☆	☆	☆	–	–	6
Nakagiri	☆	☆	☆	☆	☆	☆	☆	☆	8
Ohta	☆	☆	☆	☆	☆	☆	☆	☆	8
Prenzel	☆	☆	☆	☆	☆	☆	–	–	6
Riquet	☆	☆	☆	☆	☆	☆	☆	☆	8
Sonobe	☆	☆	–	☆	☆	☆	☆	☆	7
Tanaka	☆	☆	☆	☆	☆	☆	☆	☆	8
Tomina	☆	☆	☆	☆	☆	☆	–	–	6
Tomizawa	☆	☆	☆	☆	☆	☆	☆	☆	8
Wang	☆	☆	☆	☆	☆	☆	☆	–	7
Wang	☆	☆	☆	☆	☆	☆	–	–	6
Wang	☆	☆	☆	–	☆	☆	–	–	5
Yoshino	☆	☆	☆	☆	☆	☆	–	–	6
Zhang	☆	☆	☆	☆	☆	☆	–	–	6
Zhao	☆	☆	☆	☆	☆	☆	☆	–	7
Zheng	☆	☆	☆	☆	–	☆	☆	–	6

Risk of bias was evaluated with use of the Newcastle-Ottawa Scale in cohort studies. A score of 7 or higher indicates a low risk of bias

### Primary outcome measures

**Fig. 2:**
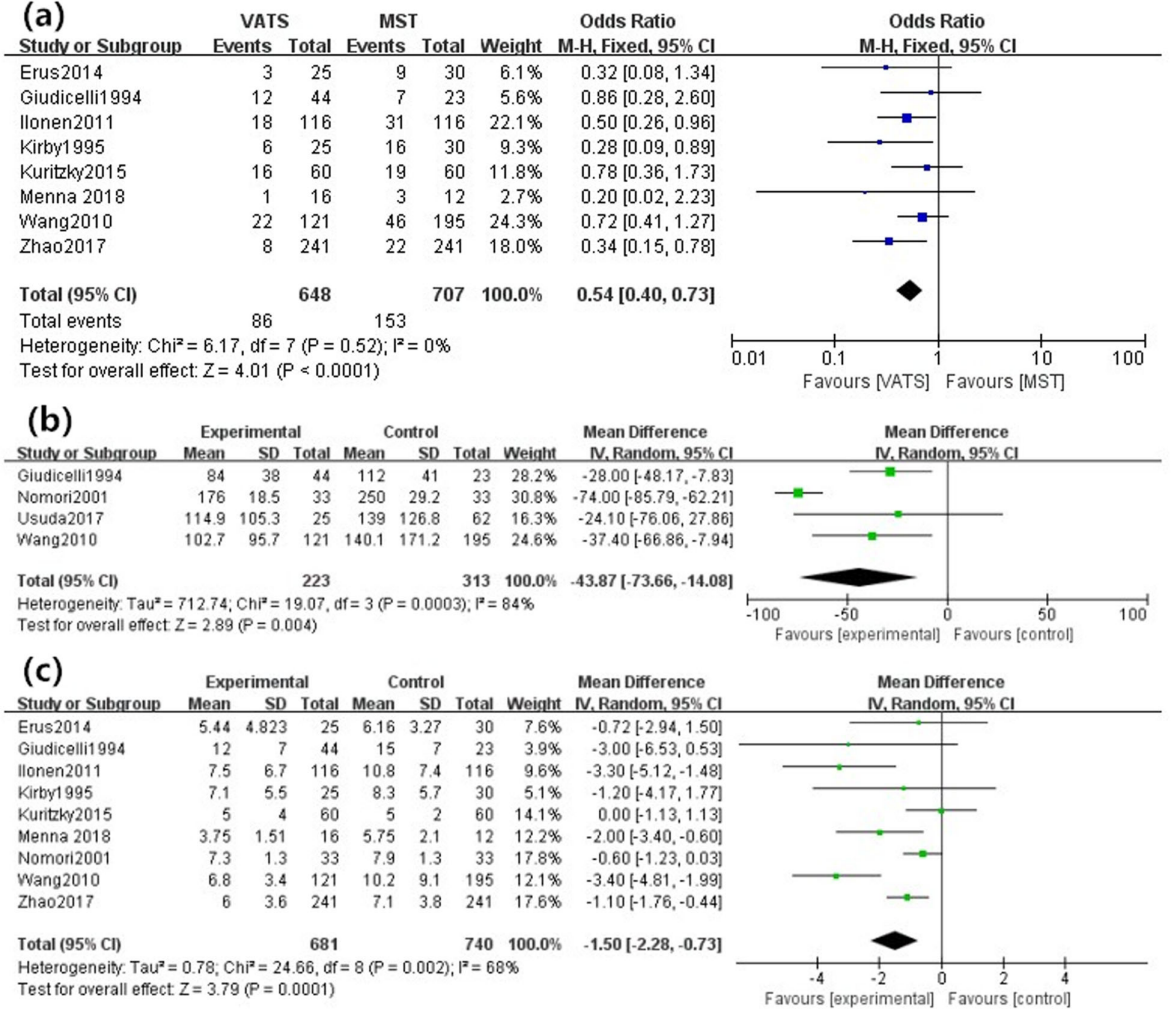
Primary outcome measurements. **a** Postoperative complications, **b** Intraoperative blood loss, **c** Hospital stay

**Fig. 3:**
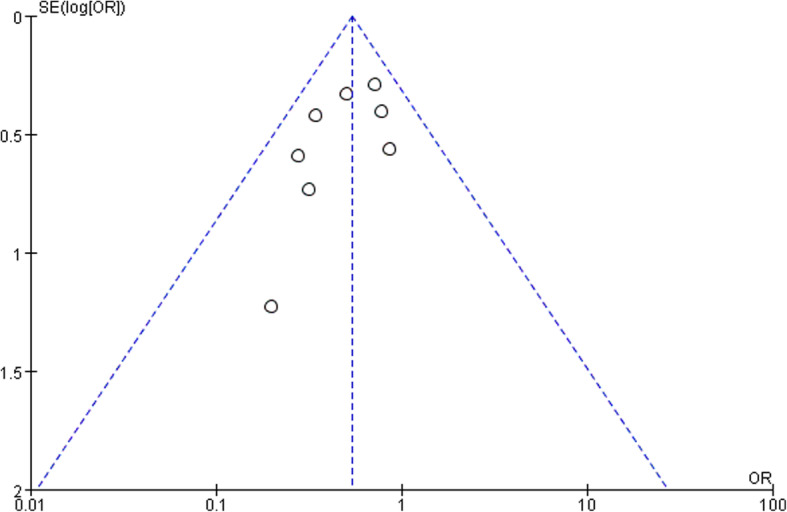
Funnel plot of complication

Eight studies [17–22, 24, 25] focused on postoperative complications assessing the safety of surgeries. A total of 86 patients in VATS group and 153 patients in MST group had postoperative complications. The pooled analysis showed that a significant reduction in the incidence of total postoperative complications [OR: 0.54, 95%CI (0.40, 0.73), *P* < 0.0001] was found in VATS group (Fig. 2a). The result was stable with no heterogeneity (I^2^ = 0, *P* = 0.52) or publication bias found among them. The funnel plot was shown in Fig. 3.

A reduction of intraoperative blood loss in VATS group was reported in 4 studies including 536 patients. There was a significant decrease [MD: -43.87, 95%CI (− 73.66, − 14.08), *P* = 0.004] in VATS group comparing with MST group (Fig. 2b).

A meta-analysis of 9 studies [16–22, 24, 25] with 1421 patients showed an obvious shortened length of hospital stay [MD: -1.50, 95%CI (− 2.28, − 0.73), *P* = 0.0001] following the VATS. Similarly, the shorten duration of chest tube drainage [MD: -0.71, 95%CI (− 1.18, − 0.24), *P* = 0.003] in VATS group was found in 6 studies [16]^,^ [18–20, 22, 24] with 845 patients enrolled. Figure 2c described detailed outcomes as well.

### Secondary outcome measures

When regarding operative time, a total of 1276 patients in 9 studies were observed, including 1 RCT and 8 cohort studies. A significant difference was discovered between 2 groups [MD: 17.11, 95%CI (2.38, 31.85), *P* = 0.02] (Table 3). The result was stable and a high heterogeneity was observed (I^2^ = 92%). However, in the included RCT, no significant difference was shown in operative time between two groups.

**Table 3:** Secondary outcome measurements

Outcomes	No. of studies	Pooling model	Effect size	95%CI		P	I^2^
Operative time	9	Random	17.11	2.38	31.85	0.02	92%
No. of resected lymph nodes	3	Random	1.25	−4.57	7.07	0.67	95%
Mortality	5	Fixed	1.03	0.38	2.8	0.95	0
Pneumonia	4	Fixed	1.11	0.48	2.55	0.8	0
Prolonged air leak	5	Random	0.79	0.32	1.93	0.6	55%
Arrythmia	5	Fixed	0.48	0.21	1.06	0.07	0
Postoperative bleeding	4	Fixed	0.62	0.24	1.61	0.32	15%
Chylothorax	4	Fixed	0.52	0.20	1.33	0.17	15%
Duration of chest tube drainage	6	Random	−0.71	−1.18	−0.24	0.003	57%
Postoperative pain in day 1	2	Fixed	−1.02	−1.37	−0.66	0.00001	0
Postoperative pain in day2	2	Fixed	−1.42	−1.75	−1.09	0.00001	0

*visual analogue scale was validated in pain assessment

As for the number of resected lymph nodes, no remarkable difference was found between two groups in the 3 studies with 864 patients involved. The result showed that a comparable lymph node retrieval rate between the two groups [MD:1.25, 95%CI(− 4.57, 7.07), *P* = 0.67]. The detailed results of number of resected lymph nodes were presented in Table 3.

Five articles discussed the postoperative mortality between two groups. However, there was no remarkable differences found in respect of postoperative mortality [OR: 1.03, 95%CI (0.38, 2.80), *P* = 0.95]. Detailed information can be found in Table 3.

### Sensitivity and publication bias

Sensitivity analysis was applied by sequentially removing all included articles to find the source of heterogeneity and the results were stable among all included articles. Publication bias was assessed by Review Manager V5.3 and a funnel plot for the analysis of complication rate was shown in Fig. 3.

## Discussion

Minimal invasive surgery has been widely accepted in lung cancer patients especially in early stage cases. VATS has been shown to be a favorable option with less operative bleeding, shorter hospital stay and less postoperative complications [26, 27]. Accumulating amount of trials focused on the comparison between VATS and conventional thoracotomy have been published. However, few studies focused on the direct comparison between less invasive muscle-sparing thoracotomy and VATS. Only one RCT have been conducted in this field and high-quality research was limited. Our work gave a better understanding and a protocol for clinical decision and future research.

This is the first systematic review and meta-analysis designed to assess the effectiveness of VATS and MST on operable lung cancer patients. The present study indicated that VATS might be superior to the MST for less operative trauma and earlier recovery reflected by the reduced amount of intraoperative blood loss, lower rate of postoperative complications, shortened length of hospital stay and chest tube drainage. In respect of postoperative mortality, little difference was observed between VATS and MST, but this part of conclusion should be interpreted with caution for limited quantity and quality of enrolled trials.

The favorable effects following VATS support the theory that minimally invasive surgery might play an essential role of smaller intraoperative injuries and fast postoperative recovery. Because of its minimal invasiveness and less chest wall trauma, VATS approach is of particular advantage to patients especially the elder ones [28]. What’s more, VATS also leads to lower VAS scoring [29], which is essential for postoperative recovery. Conventional surgical approach is usually associated with a decline in pulmonary function. While after VATS approach, chemotherapy is generally better tolerated than after conventional thoracotomy, as is shown by multiple trials [30, 31]. Another important merit of VATS is that it is even more beneficial those with limited pulmonary function, which make itself more adaptive to the treatment of lung cancer with chronic obstructive pulmonary disease patients [32, 33]. A recent study discussed the inflammation differences between the two group (VATS and MST) and a reduced inflammation was found during minimal invasive surgery both locally and systematically [34]. In contrast to common view, VATS represents higher cost, many studies have reported a reduction of medical cost with VATS approach [35, 36], probably because of shortening the length of hospital stay and reducing healthcare resources utilization. According to a recent report published by a panel of 55 experts in VATS lobectomy, VATS approach should be the standard of care for the resection of early-stage lung cancer except in certain clinical situations [37].

There are still few limitations in our study. It was difficult to stratify potential cofounders such as age, stage of cancer, which are closely associated with pulmonary function. Furthermore, the methodological quality of some included studies was not sufficient, which might disturb analysis outcomes. Finally, the experience of surgeon and institution were inconsistent among included studies, which may contribute to the high heterogeneity.

## Conclusion

In conclusion, this present systematic review and meta-analysis indicates that the benefit of VATS was superior to that of MST in the reduction of intraoperative trauma, postoperative complications and enhanced recovery after surgery for patients with operable lung cancer. In consideration of some limitations of enrolled studies, the conclusion should be carefully applied. Further high-quality randomized controlled trials are needed.

## Data Availability

All data generated or analyzed during this study are included in this published article.

## Abbreviations

95% CI: 95% confidence intervals
MD: Mean difference
MST: Muscle-sparing thoracotomy
NSCLC: Non-small cell lung cancer
OR: Odds ratios
VATS: Video-assisted thoracoscopic surgery
